# Low prevalence of *Blastocystis* sp. in active ulcerative colitis patients

**DOI:** 10.1007/s10096-015-2312-2

**Published:** 2015-02-14

**Authors:** N. G. Rossen, A. Bart, N. Verhaar, E. van Nood, R. Kootte, P. F. de Groot, G. R. D’Haens, C. Y. Ponsioen, T. van Gool

**Affiliations:** 1 Department of Gastroenterology and Hepatology, Academic Medical Center, Amsterdam, The Netherlands; 2 Department of Medical Microbiology, Parasitology Section, Academic Medical Center, Amsterdam, The Netherlands; 3 Department of Internal Medicine, Academic Medical Center, Amsterdam, The Netherlands; 4 Department of Vascular Medicine, Academic Medical Center, Amsterdam, The Netherlands

## Abstract

Ulcerative colitis (UC) is thought to originate from a disbalance in the interplay between the gut microbiota and the innate and adaptive immune system. Apart from the bacterial microbiota, there might be other organisms, such as parasites or viruses, that could play a role in the aetiology of UC. The primary objective of this study was to compare the prevalence of *Blastocystis* sp. in a cohort of patients with active UC and compare that to the prevalence in healthy controls. We studied patients with active UC confirmed by endoscopy included in a randomised prospective trial on the faecal transplantation for UC. A cohort of healthy subjects who served as donors in randomised trials on faecal transplantation were controls. Healthy subjects did not have gastrointestinal symptoms and were extensively screened for infectious diseases by a screenings questionnaire, extensive serologic assessment for viruses and stool analysis. Potential parasitic infections such as *Blastocystis* were diagnosed with the triple faeces test (TFT). The prevalence of *Blastocystis* sp. were compared between groups by Chi-square testing. A total of 168 subjects were included, of whom 45 had active UC [median age 39.0 years, interquartile range (IQR) 32.5–49.0, 49 % male] and 123 were healthy subjects (median age 27 years, IQR 22.0–37.0, 54 % male). *Blastocystis* sp. was present in the faeces of 40/123 (32.5 %) healthy subjects and 6/45 (13.3 %) UC patients (*p* = 0.014). Infection with *Blastocystis* is significantly less frequent in UC patients as compared to healthy controls.

## Introduction

The microbiota are believed to play an important role in the interaction between the host and the immune system. Commensal microbes are believed to protect our body against invading pathogens and have an important function in the development of the mucosal immune system [1]. Ulcerative colitis (UC) is thought to originate from an uncontrolled mucosal immune response initiated by luminal microbiota. An aberrant microbiome in UC has been described in several studies, characterised by reduced numbers of *Firmicutes* and *Bacteroidetes*, as well as a lower species diversity as compared to controls [2–4]. Besides bacteria however, the gut is also colonised by viruses (5.8 %), archaea (0.8 %) and eukaryotes (0.5 %) [5], although little is known about the relevance of these microorganisms in UC. One of the eukaryotes that is still debated with regard to its pathogenic role is *Blastocystis* sp.. In the literature, *Blastocystis* prevalence in asymptomatic or symptomatic subjects varies from 0.5 % in Japan up to 100 % observed in children living in villages of the Senegal River Basin [6, 7]. The prevalence of *Blastocystis* sp. was studied in patients with irritable bowel syndrome (IBS) for a possible causative relation with abdominal symptoms and the presence of *Blastocystis* [8, 9]. In inflammatory bowel disease (IBD), the question remains as to whether eradication of an infection with *Blastocystis* would relieve abdominal symptoms such as abdominal pain or bloating [10, 11]. Conflicting data exist on the role of this eukaryote in either disease [12, 13]. Recently, a study was performed on *Blastocystis* infection and the association with the microbiota. Interestingly, *Blastocystis* was found to be associated with a decrease of faecal microbiota protective bacteria, such as *Bifidobacterium* sp. and *Faecalibacterium prausnitzii* [8].

The aim of our study was to compare the prevalence of *Blastocystis* sp. in a well-defined cohort of patients with active UC to a cohort of healthy controls.

## Materials and methods

### UC patients and healthy volunteers

Patients with active UC participating in a clinical trial between 23-5-2011 and 30-06-2013 were included if they had an exacerbation of their disease, defined by a Simple Clinical Colitis Activity Index (SCCAI) score of ≥4 and ≤11 [14]. Endoscopic activity was confirmed by an endoscopic Mayo score of ≥1. Patients were allowed to use a stable dose of medication for UC, except for biologicals or corticosteroids >10 mg per day, and had not used antibiotics in the 6 weeks before inclusion. Healthy volunteers were involved in one of the faecal microbiota transplantation (FMT) trials at our study centre from 2008 to 2013. They participated as faecal donors and were regarded as a control cohort. We collected all clinical and screening data. These volunteers were screened for faecal donation in either a clinical faecal transplantation trial on FMT in UC, type I diabetes, type II diabetes/metabolic syndrome or for patients suffering from recurrent *Clostridium difficile* infection [15]. Healthy volunteers were relatives of the patients or unrelated volunteers working or studying at the university hospital centre and were required to be completely free of gastrointestinal symptoms. They were extensively interviewed on sexual risk behaviour and recent travel behaviour by a detailed questionnaire. Donors were excluded if they had travelled to a developing country within 6 months before screening or if they were suspected of sexual risk behaviour. Use of antibiotics in the previous 8 weeks was also considered an exclusion criterium. A gastrointestinal infection was ruled out by faecal tests on pathogenic bacteria, parasites and viruses [enteropathogenic bacteria: *Salmonella*, *Shigella*, *Yersinia enterocolitica* and *Campylobacter*, evaluated by local standards, *Clostridium difficile*, evaluated by toxin enzyme-linked immunosorbent assay (ELISA) and culture; enteropathogenic parasites, including *Blastocystis* sp. and *Dientamoeba fragilis*, evaluated by the triple faeces test (TFT), as described below; viruses, including norovirus, rotavirus, astrovirus, adenovirus, enterovirus, parechovirus and sapovirus, evaluated by real-time TaqMan polymerase chain reaction (PCR) using the Roche LightCycler 480 instrument [16]] in both healthy volunteers and UC patients.

The study protocol was approved by the Medical Ethical Committee of the Academic Medical Center in Amsterdam, the Netherlands (METC registry number #2011_005). All subjects signed an informed consent form.

### Faecal tests on *Blastocystis* sp

The TFT was used for the diagnosis of intestinal parasites, including *Blastocystis* sp. [17, 18]. In this test stool, samples of a patient are collected in three tubes on three consecutive days before delivery in the hospital. In two of the three tubes, on day 1 and day 3, a preservation sodium acetate–acetic acid–formalin (SAF) solution is present, with which vegetative stages of protozoa, including *Blastocystis* sp., are well preserved. With the tube from the second day, a fresh, non-conserved, stool sample is collected. Samples which are fixed in SAF are examined with wet smears and chlorazol black permanent stain. Fresh stool from the second day is concentrated according to Ridley and the sediment microscopically examined for cysts of protozoa and eggs of helminths. The results of microscopic examinations of all three samples are combined to provide the final result of the TFT test of the patient. As diagnostic criterion for positivity of a sample for *Blastocystis* sp., at least two clear vacuolar forms in either of the two SAF-fixed samples had to be present. Granular or amoeboid stages of *Blastocystis* sp. were not studied. As diagnostic criteria for positivity of a sample for *Dientamoeba fragilis*, at least two clear vacuolar forms in either of the two SAF-fixed samples had to be present. The diagnosis was confirmed by a chlorazol black dye permanent stain.

### Statistical analysis

Based on an expected absolute difference in prevalence rates of 30 % (30 % vs. 60 %), a desired power of 80 % and a two-sided α-level of 0.05, 50 subjects per group were calculated for primary analyses. Data were expressed as the mean and 95 % confidence interval (CI) of the mean or, when they were not normally distributed, as the median and interquartile range (IQR). The prevalence rates of *Blastocystis* sp., diagnosed by the TFT, between study groups were compared by Chi-square testing if the expected cell size was >5, and by Fisher’s exact test in case of smaller cell sizes. A *p*-value <0.05 was considered statistically significant. Statistical analyses were performed using SPSS version 21.0 software (SPSS Inc., Chicago, IL, USA).

## Results

Forty-five UC patients and 123 healthy subjects were studied. The recruitment periods were randomly distributed between summer and winter for both groups. Forty-four percent of UC patients (*n* = 20) and 50 % of healthy subjects (*n* = 62) were included from the winter period (October–March). Forty-six percent of UC patients (*n* = 25) and 50 % of healthy subjects were included during summer (*n* = 61). Gender was equally distributed across groups. Healthy subjects were younger than the included UC patients (*p* < 0.001). The median disease duration of UC was 9 years (IQR 0–27). According to the Montreal classification, UC patients were classified as: proctitis (*n* = 3, 6.7 %), left-sided colitis (*n* = 24, 53.3 %) or pancolitis (*n* = 18, 40.0 %), as the most severe ever documented. In all UC patients, mucosal disease activity was confirmed by endoscopy. Seven patients (15.6 %) had an active proctitis, 28 (62.2 %) had a left-sided colitis and 10 (22.2 %) had a pancolitis. The median SCCAI score, assessing clinical activity, was 8 (IQR 4–11). None of the included healthy subjects or UC patients turned out to have an infection with bacterial, viral or parasitic pathogens. The characteristics of the study population and medication use at inclusion are shown in Table 1.

**Table 1:** Characteristics of the study population

	UC patients, *n* = 45	Healthy controls, *n* = 123	*p*-Value
Male, *n* (%)	22 (49)	67 (54)	0.601
Proctitis, *n* (%)	3 (6.7)	−	n/a
Left-sided colitis, *n* (%)	24 (53.3)	−	n/a
Pancolitis, *n* (%)	18 (40.0)	−	n/a
Age, years (median, IQR)	39 (32.5–49.0)	27 (22.0–37.0)	<0.001
Disease duration, years (median, IQR)	9 (0–27)	−	n/a
Medication, *n* (%)	29 (64.4 %)	0 (0)	n/a
Mesalamine oral, *n* (%)	29 (64.4 %)	−	n/a
Mesalamine/corticosteroids rectal, *n* (%)	13 (28.9 %)	−	n/a
Immunosuppressants (thiopurine), *n* (%)	13 (28.9 %)	−	n/a
Corticosteroids, systemic, *n* (%)	9 (20.0 %)	−	n/a

### Prevalence of *Blastocystis* sp. in UC patients and healthy controls

Six of the 45 UC patients (13 %) had a positive TFT test for *Blastocystis* sp. versus 40 of the 123 (33 %) healthy controls (*p* = 0.014) (Table 2). *Blastocystis* sp. infection was equally distributed across UC patients with proctitis, left-sided colitis and pancolitis (Table 2), *p* = 0.20. None of the treatments that the UC patients were receiving for their UC (mesalamine, immunosuppressants, corticosteroids) was associated with higher infection rates with *Blastocystis* sp. (data not shown). No significant differences were found when comparing the proportion of infected female subjects [vs. the proportion of infected male patients per group (Table 2)], *p* = 0.41 for UC patients and *p* = 0.64 for healthy controls. In a multivariate logistic regression analysis corrected for age, healthy controls had a 2.86 odds ratio (95 % CI 1.09–7.53, *p* = 0.03) for *Blastocystis* infection compared to UC patients.

**Table 2:** Prevalence of *Blastocystis* sp. for ulcerative colitis (UC) patients and healthy controls

	UC patients, *n* = 45	Healthy controls, *n* = 123	*p*-Value
Positive for *Blastocystis* sp., *n* (%)	6 (13)	40 (33)	0.014*
Male, *n* (%)	2 (33)	23 (58)	0.38**
Proctitis, *n* (%)	1 (2)	−	
Left-sided colitis, *n* (%)	4 (9)	−	
Pancolitis, *n* (%)	1 (2)	−	

*Chi-square test

**Fisher’s exact probability test

Other intestinal protozoa observed in the UC population included *Dientamoeba fragilis*. There was no significant difference in the prevalence of *Dientamoeba fragilis* between UC patients [6 out of 45 (13 %) were found to be positive] and healthy controls [27 out of 123 (22 %) were found to be positive], *p* = 0.21.

## Discussion

This study reports on the prevalence of *Blastocystis* sp. in patients with clinical and endoscopic active UC compared to a well-defined cohort of healthy controls, determined by the use of the TFT. Infection with *Blastocystis* sp. was significantly less frequent (13.3 %) in UC patients compared to healthy controls (32.5 %), *p* = 0.014.

Health is a status which is hard to define without specific markers available. We compared patients with active UC and healthy controls using strict predefined definitions of the studied subjects. Healthy controls were extensively questioned on any risk for infection due to sexual risk behaviour or travelling to developing countries. Only if they were not suspect for any risk behaviour were they selected for faecal and serologic screening on pathogens (either bacterial, viral or parasitic). In UC patients, infectious causes of an exacerbation of disease were ruled out according to the same screening procedure. None of the included healthy subjects or UC patients turned out to have an infection with bacterial, viral or parasitic pathogens. In the available literature on *Blastocystis* sp., ‘healthy subjects’ were not characterised by the absence of infectious diseases or could be on chronic medication. Scanlan et al. performed a study on *Blastocystis* prevalence in a group of 105 healthy individuals comparable to the current sample size of 123 healthy controls [19]. They were not reported to be symptomatic or not. Besides that, subjects >65 years of age derived from a hospital setting will not be the optimal ‘healthy’ population to study. In the current study, strict definition of both study groups made possible a clear comparison of the true prevalence of *Blastocystis* sp. in both UC patients and healthy controls. Whereas we did not find medication to be associated with the presence or absence of *Blastocystis* sp. in UC patients, it is hard to exclude any role for medication that was only used in UC patients and not by subjects in the control group. Both the TFT and PCR are highly reliable for the diagnosis of *Blastocystis* sp. Microscopy by the TFT was slightly superior in terms of sensitivity than PCR as tested in the stools of 107 patients [18].

When comparing the current study to previously published data, it is important to take into account that there are several factors potentially influencing the prevalence of *Blastocystis* infections. Examples are the indication for the test: studying symptomatic patients or asymptomatic subjects; preservation of faecal material; and the diagnostic procedures used for parasite testing (PCR or TFT). Even by using the same PCR technique for the detection of *Blastocystis* sp., the sensitivity is dependent on the commercial DNA extraction kit used [20]. Also, among microscopy procedures, conventional staining procedures such as Lugol’s stain and trichrome staining showed lower sensitivity compared to immunofluorescence antibody (IFA) staining using an immunofluorescent antibody for *Blastocystis* [21]. Age was studied to be of influence on the incidence of *Blastocystis* infection. A study performed on healthcare-seeking patients in Karachi showed an age distribution with a relatively high prevalence at middle age compared to young adults [22]. Older age was found to be associated with *Blastocystis* infection as observed in primary care patients diagnosed with IBS and aged 18–50 years from Denmark [23]. This observation was also found in an Australian population of patients positive for an enteric protozoan infection [24]. A comparable prevalence of *Blastocystis* sp. was found in healthy subjects >65 years of age to subjects <65 years of age, as described by Scanlan at al [19]. The association of age and *Blastocystis* was not well studied in UC patients. In our study, UC patients were older than healthy controls. Multivariate analysis revealed that age was not a predictor for *Blastocystis* positivity. If older age would be associated with a higher prevalence of *Blastocystis* sp. also in UC patients, the observed difference in prevalence would be even greater if both study groups were age-matched. Furthermore, environmental factors such as hygienic circumstances and the geographical location under study play a role in the outcome of prevalence studies on *Blastocystis* sp.

In a case–control study comparing the prevalence of *Blastocystis* sp. by applying native Lugol and formol-ethyl acetate concentration to stool specimens in patients with IBD and IBS as opposed to asymptomatic healthy controls, a higher prevalence of *Blastocystis* sp. was detected in UC patients [13]. Conversely, a study performed by Petersen et al., in which a PCR was used to diagnose *Blastocystis*, suggested that active UC patients showed a lower prevalence of *Blastocystis* sp. and *Dientamoeba fragilis* infection compared to healthy controls [25].

Relatively high prevalence rates of *Blastocystis* infection (32.5 %) were observed in our healthy control group. In comparison to a study we performed on 442 patient samples from routine clinical parasitology, 24.0 % tested positive with the TFT for *Blastocystis* sp. [18], which is lower than the observed prevalence of 32.5 % in the current cohort of healthy controls. Of note, the prevalences in both the healthy control group and UC patients in the present study are higher than those observed by Petersen et al. (healthy controls: 32.5 % by TFT vs. 19 % with PCR in two cohorts of medical and laboratory staff and recruits; UC patients: 13 % by TFT vs. 0 % with PCR with Petersen et al. [25]). Nevertheless, one should be cautious when comparing data measured by different diagnostic approaches.

All UC patients in our study had an exacerbation of their disease. Prevalence rates were not compared with a UC cohort of patients in remission. Petersen et al. observed that *Blastocystis* infection was significantly less prevalent in UC patients with active disease compared to patients with inactive disease. This phenomenon was not observed in active and inactive Crohn’s disease patients [25].

In our study, we found a lower prevalence of *Blastocystis* measured by a highly sensitive technique such as microscopy in UC patients as compared to a large cohort of healthy subjects. This observation supports the hygiene hypothesis suggesting that the increase in chronic inflammatory disorders is at least partly attributable to immune dysregulation. This might result from the lack of exposure to microorganisms that help to establish the ‘normal’ background levels of immune regulation [26]. From mouse models, we learned that germ-free animals fail to develop spontaneous colitis [27]. There is growing evidence that disturbances in the bacterial microbiota result in immunological dysregulation that may underlie disorders such as IBD [28]. Alternatively, *Blastocystis* sp. may be more sensitive to the inflammatory conditions in UC patients, leading to the parasite being cleared by UC patients more often than by healthy controls. Hopefully, metagenomic approaches will not only yield more insight into the role of the microbiota in health and disease, but may also elucidate the complex network of microorganisms being part of the gastrointestinal tract.

The pathogenicity of *Blastocystis* has been debated multiple times, but no consensus has been achieved as to whether this organism is related to intestinal symptoms such as nausea, abdominal pain and diarrhoea [29–31]. We did not aim to answer any question regarding the relationship between the presence of *Blastocystis* sp. and intestinal symptoms in UC patients in the current study. Notwithstanding, the high prevalence observed in asymptomatic healthy controls points to a commensal immunoregulatory role for these parasites. As aberrancies in the intestinal microbiota are found in UC patients, it still remains unknown whether this is a cause or a consequence of chronic inflammation. From this perspective, it would be interesting to investigate whether the observed low prevalence of *Blastocystis* sp. was present already during childhood and may persist after UC diagnosis. FMT with faeces from a healthy donor can potentially restore the low-diversity faecal microbiota in UC. Future studies should investigate whether the occurrence of *Blastocystis* increases after FMT. FMT would be an interesting model to investigate whether microbiota changes come along with a different community attractive for *Blastocystis* sp.

## References

[1] Rakoff-Nahoum S, Paglino J, Eslami-Varzaneh F, Edberg S, Medzhitov R (2004). Recognition of commensal microflora by toll-like receptors is required for intestinal homeostasis. Cell.

[2] Rajilić-Stojanović M, Shanahan F, Guarner F, de Vos WM (2013). Phylogenetic analysis of dysbiosis in ulcerative colitis during remission. Inflamm Bowel Dis.

[3] Frank DN, Robertson CE, Hamm CM, Kpadeh Z, Zhang T, Chen H (2011). Disease phenotype and genotype are associated with shifts in intestinal-associated microbiota in inflammatory bowel diseases. Inflamm Bowel Dis.

[4] Machiels K, Joossens M, Sabino J, De Preter V, Arijs I, Eeckhaut V (2014). A decrease of the butyrate-producing species *Roseburia hominis* and *Faecalibacterium prausnitzii* defines dysbiosis in patients with ulcerative colitis. Gut.

[5] Arumugam M, Raes J, Pelletier E, Le Paslier D, Yamada T, Mende DR (2011). Enterotypes of the human gut microbiome. Nature.

[6] Horiki N, Maruyama M, Fujita Y, Yonekura T, Minato S, Kaneda Y (1997). Epidemiologic survey of *Blastocystis hominis* infection in Japan. Am J Trop Med Hyg.

[7] El Safadi D, Gaayeb L, Meloni D, Cian A, Poirier P, Wawrzyniak I (2014). Children of Senegal River Basin show the highest prevalence of *Blastocystis* sp. ever observed worldwide. BMC Infect Dis.

[8] Nourrisson C, Scanzi J, Pereira B, NkoudMongo C, Wawrzyniak I, Cian A (2014). *Blastocystis* is associated with decrease of fecal microbiota protective bacteria: comparative analysis between patients with irritable bowel syndrome and control subjects. PLoS One.

[9] Poirier P, Wawrzyniak I, Vivarès CP, Delbac F, El Alaoui H (2012). New insights into *Blastocystis* spp.: a potential link with irritable bowel syndrome. PLoS Pathog.

[10] Yamamoto-Furusho JK, Torijano-Carrera E (2010). Intestinal protozoa infections among patients with ulcerative colitis: prevalence and impact on clinical disease course. Digestion.

[11] Nagler J, Brown M, Soave R (1993). *Blastocystis hominis* in inflammatory bowel disease. J Clin Gastroenterol.

[12] Krogsgaard LR, Engsbro AL, Stensvold CR, Nielsen HV, Bytzer P (2014) The prevalence of intestinal parasites is not greater among individuals with irritable bowel syndrome: a population-based case–control study. Clin Gastroenterol Hepatol. doi: 10.1016/j.cgh.2014.07.065 10.1016/j.cgh.2014.07.065 25229421

[13] Cekin AH, Cekin Y, Adakan Y, Tasdemir E, Koclar FG, Yolcular BO (2012). Blastocystosis in patients with gastrointestinal symptoms: a case–control study. BMC Gastroenterol.

[14] Walmsley RS, Ayres RC, Pounder RE, Allan RN (1998). A simple clinical colitis activity index. Gut.

[15] van Nood E, Vrieze A, Nieuwdorp M, Fuentes S, Zoetendal EG, de Vos WM (2013). Duodenal infusion of donor feces for recurrent *Clostridium difficile*. N Engl J Med.

[16] Molenkamp R, van der Ham A, Schinkel J, Beld M (2007). Simultaneous detection of five different DNA targets by real-time Taqman PCR using the Roche LightCycler480: application in viral molecular diagnostics. J Virol Methods.

[17] van Gool T, Weijts R, Lommerse E, Mank TG (2003). Triple Faeces Test: an effective tool for detection of intestinal parasites in routine clinical practice. Eur J Clin Microbiol Infect Dis.

[18] Bart A, Wentink-Bonnema EMS, Gilis H, Verhaar N, Wassenaar CJA, van Vugt M (2013). Diagnosis and subtype analysis of *Blastocystis* sp. in 442 patients in a hospital setting in the Netherlands. BMC Infect Dis.

[19] Scanlan PD, Stensvold CR, Rajilić-Stojanović M, Heilig HGHJ, De Vos WM, O’Toole PW (2014). The microbial eukaryote *Blastocystis* is a prevalent and diverse member of the healthy human gut microbiota. FEMS Microbiol Ecol.

[20] Yoshikawa H, Dogruman-Al F, Turk S, Kustimur S, Balaban N, Sultan N (2011). Evaluation of DNA extraction kits for molecular diagnosis of human *Blastocystis* subtypes from fecal samples. Parasitol Res.

[21] Dogruman-Al F, Simsek Z, Boorom K, Ekici E, Sahin M, Tuncer C (2010). Comparison of methods for detection of *Blastocystis* infection in routinely submitted stool samples, and also in IBS/IBD Patients in Ankara, Turkey. PLoS One.

[22] Haider SS, Baqai R, Qureshi FM, Boorom K (2012). *Blastocystis* spp., *Cryptosporidium* spp., and *Entamoeba histolytica* exhibit similar symptomatic and epidemiological patterns in healthcare-seeking patients in Karachi. Parasitol Res.

[23] Engsbro AL, Stensvold CR, Vedel Nielsen H, Bytzer P (2014). Prevalence, incidence, and risk factors of intestinal parasites in Danish primary care patients with irritable bowel syndrome. Scand J Infect Dis.

[24] Fletcher S, Caprarelli G, Merif J, Andresen D, Van Hal S, Stark D (2014). Epidemiology and geographical distribution of enteric protozoan infections in Sydney, Australia. J Public Health Res.

[25] Petersen AM, Stensvold CR, Mirsepasi H, Engberg J, Friis-Møller A, Porsbo LJ (2013). Active ulcerative colitis associated with low prevalence of *Blastocystis* and *Dientamoeba fragilis* infection. Scand J Gastroenterol.

[26] Rook GAW (2011). Hygiene and other early childhood influences on the subsequent function of the immune system. Dig Dis.

[27] Sellon RK, Tonkonogy S, Schultz M, Dieleman LA, Grenther W, Balish E (1998). Resident enteric bacteria are necessary for development of spontaneous colitis and immune system activation in interleukin-10-deficient mice. Infect Immun.

[28] Round JL, Mazmanian SK (2009). The gut microbiota shapes intestinal immune responses during health and disease. Nat Rev Immunol.

[29] Kain KC, Noble MA, Freeman HJ, Barteluk RL (1987). Epidemiology and clinical features associated with *Blastocystis hominis* infection. Diagn Microbiol Infect Dis.

[30] Leder K, Hellard ME, Sinclair MI, Fairley CK, Wolfe R (2005). No correlation between clinical symptoms and *Blastocystis hominis* in immunocompetent individuals. J Gastroenterol Hepatol.

[31] Udkow MP, Markell EK (1993). *Blastocystis hominis*: prevalence in asymptomatic versus symptomatic hosts. J Infect Dis.

